# Breast Cancer Risk Perception and Mammography Screening Behavior of Women in Northeast Brazil

**DOI:** 10.1089/whr.2019.0026

**Published:** 2020-06-02

**Authors:** Saionara Açucena Vieira Alves, Mathias Weller

**Affiliations:** Post Graduate Program in Public Health, State University of Paraíba (UEPB), Campina Grande, Paraíba, Brazil.

**Keywords:** breast cancer, perceived risk, mammography screening behavior

## Abstract

***Background:*** Previous studies suggest that education and income affect Brazilian women's breast cancer prevention behavior. The present study focused on the impact of perceived and estimated risk on mammography screening (MS) behavior.

***Materials and Methods:*** Information regarding socioeconomic variables and risk perception was obtained from 396 healthy women aged 40–79 years. Perceived comparative risk was measured on a seven-point Likert scale. A Breast Cancer Risk Assessment Tool of 5-year risk to develop breast cancer was used to determine objective risk. Estimated comparative risk was determined as categories of perceived risk relative to the objective risk. Regression analysis was applied to determine odds ratios (ORs) and confidence intervals (95% CIs) of variables.

***Results:*** Asked about the potential of MS to lower risk of death because of breast cancer, 215 (54.29%) responded that it does not lower risk. Women with low perceived comparative risk had a twofold (OR = 0.493; 95% CI: 0.24–1.00) decreased chance to participate in MS annually, compared with women with high-perceived comparative risk (*p* = 0.020). Women without family history had a 7.6-fold (OR = 0.132; 95% CI: 0.07–0.25) decreased chance of having a high-perceived comparative risk (*p* = 0.000). If compared with underestimation, the overestimation and accurate estimation of comparative risk tended to be associated with a decreased chance of MS attendance (*p* = 0.017). Regression modeling indicated that low educational level, no occupation, and no family history decreased the chance of women having MS (*p* = 0.040; *p* = 0.010; *p* = 0.022).

***Conclusions:*** Risk perception depended on family history. Present data did not indicate that overestimation, or accurate estimation of comparative risk, increased chance of MS attendance. Educational level, occupation status, and family history, instead, determined MS performance.

## Introduction

Incidence and mortality rates of breast cancer are stable and have even declined in several developed countries of Europe and North America.^1^ By contrast, in developing countries in Asia, Africa, and South America, mortality rates are increasing.^1^

In Brazil, the largest Latin American country, the National Cancer Institute (INCA) predicted 66.280 new breast cancer cases for 2021.^2^ In the southern regions of Brazil, including urban centers such as São Paulo and Rio de Janeiro, breast cancer incidence has remained stable or declined in recent years.^2,3^ The Northeastern region of Brazil, by contrast, has suffered an increase in breast cancer incidence: between 2005 and 2020, the incidence increased from 27.23 to 44.29 new cases per 100,000 women.^2,3^

Since 2003, Brazil has a public program for early detection of breast cancer. This is an opportunistic, not an organized, screening program, and women are not invited to participate. Furthermore, in Brazil, there are conflicting recommendations regarding the age threshold and interval for screening: the Ministry of Health recommends biannual mammography for women aged 50–69 years, whereas the Brazilian Society of Mastology recommends annual mammography screening (MS) starting at 40 years.^2,4^ For healthy women aged 70 or older, regular MS is not recommended by public health authorities.^2,3^ Therefore, for individual women, the recommendations regarding starting age and interval of MS depend mainly on the opinion of their respective physicians.

In Northeast Brazil, women often present at advanced stages of disease (Stage III and IV). High breast cancer mortality rates were attributed to nonadherence to the MS program.^5–7^ Brazilian studies identified high income as the most important predictor of adherence.^5,8,9^ Two studies also identified higher educational level as predictor of adherence to the MS program.^5,8^ Furthermore, women with first- or second-degree relatives who had any type of cancer underwent regular MS more often than did women without any cancer in their families.^9^

Perception of risk comprehension may be another variable that affects women's MS behavior. The literature distinguishes between absolute, comparative, and numeric perceived risk.^10–12^ Estimated comparative risk, generally is determined as the relationship between perceived and objective risk. The latter can be measured with various models.^13^ Several previous studies focused on women's risk estimates of breast cancer.^11,13–15^ Other studies focused on possible associations between risk estimates and women's MS behavior.^10,12,16–20^

To the best of our knowledge, there are no Brazilian studies aimed at elucidation of the possible association between risk estimates and women's MS behavior. Increasing incidence and mortality rates in Northeast Brazil highlight the importance of identifying variables that affect adherence to the MS program. We asked whether overestimation and accurate estimation of comparative risk are positively associated with women's participation on the MS program. Risk perception and estimation were analyzed in the context of other important sociodemographic variables.

## Materials and Methods

### Study population and data collection

The data sampling protocol was reviewed and approved by the Brazilian National Ethics Research Committee (CAAE Plataforma Brasil: 63089416.0.0000.5187). Written informed consent was obtained from each participant of the study. Consent to publish data anonymously was obtained from each participant of the study.

Participating women were interviewed in waiting rooms for outpatient treatment of the health service center “Dr. Francisco Pinto” and the “Hospital Municipal Dr. Edgley,” both in Campina Grande, Northeast Brazil. Both are public institutions that do not offer MS and attend mainly to low-income persons without health insurance. In contrast to the “Hospital Municipal Dr. Edgley,” the health service center “Dr. Francisco Pinto” has no inpatient treatment. No differences were found among women of both institutions. Situated in the interior, about 120 km west of the state capital João Pessoa on the Atlantic coast, Campina Grande has a population of 385,276 (2010), making it the second largest city in the state of Paraíba.^21^ Interviews were performed between March and October 2017. Socioeconomic variables and perception, respectively, estimation of breast cancer risk, were not different between women of both health service centers.

Interview of participants was always performed by one of the authors. Women were directly asked to participate. After explanation of the project and ethical considerations, the face-to-face interview started with a questionnaire filled out by one of the authors. To avoid selection bias, it was tried to interview all women present in the waiting room in the morning (8 o'clock a.m.–12 o'clock p.m.) and/or in the afternoon (2 o'clock p.m.–6 o'clock p.m.) of a defined day. Altogether, 430 women were asked to participate in the study, and 396 (92.10%) agreed in participation. Of these 396 women, 195 and 201 were from the “Hospital Municipal Dr. Edgley” and the health service center “Dr. Francisco Pinto,” respectively. Interviews normally were performed one to three times per week.

Women were eligible for the study if they were 40 years or older and had not had any type of breast or ovarian cancer. Most of the interviewed women accompanied children, mainly because of vaccination. Other ones had viral infections causing cough and sneeze, or gastrointestinal problems. Furthermore, of each group of related persons, only one woman was interviewed to avoid possible repetitive information from family members.

### Questionnaire and measures

Interviews were based on a modified structured questionnaire developed in previous studies.^9,22^ The questionnaire was subdivided into the following sections: (1) socioeconomic characteristics; (2) reproductive and health characteristics, including information regarding previous biopsies, and breast or ovarian cancer of the participant and first-degree relatives; (3) adherence to the MS program; and (4) perception of breast cancer risk.

Educational level was defined as follows. (1) Fundamental school with duration of 8 years was defined as “low.” (2) Middle school with duration of 11 years was defined as “middle.” (3) Higher educational levels were defined as “high.” Income was defined as minimum wage and multiple values of the minimum. The minimum wage in 2017 was R$937.00/month (US$184.79/month; 19th March 2020). Ethnic origin was based on self-reporting by interviewed women. Women were asked about their actual adherence to recommendations by the MS program. If asked about mammography utilization, the following options were distinguished: never, sometimes, every year, and every 2 years.

#### Objective risk

We used the Breast Cancer Risk Assessment Tool of the National Institute of Health (NIH) to calculate participants' 5-year risk of developing invasive breast cancer. The tool is based on the Gail model.^23^ Following data were used to calculate the 5-year risk with the tool. (1) Medical history of any breast cancer or ductal carcinoma *in situ*; this was for all 396 women. (2) Mutation in either the *BRCA1* or *BRCA2* gene; this was unknown in the case of all 396 women. (3) Age. (4) Race/ethnicity; this was in all 396 cases, Hispanic/Latinas born outside the United States. (5) Information about breast biopsies with benign diagnosis and their number. (6) Information about atypical hyperplasia; this was unknown in the case of all 396 women. (7) Women's age at the time of first menstrual period. (8) Women's age when they gave birth to the first child. (9) Number of first-degree relatives who had breast cancer.

The risk of all 396 women to develop invasive breast cancer within the next 5 years was on average 0.86% (standard deviation [SD] = 0.31). The 5-year risk of 167 women, aged from 40 to 49 years, varied from 0.40% to 1.30% and was on average 0.56% (SD = 0.14), whereas in the group of 229 women aged ≥50 years, it varied from 0.80% to 1.40% and was on average 1.08% (SD = 0.19; *p* = 0.000).

The tool also provides data on mean objective risk at particular ages. According to a previous study, categories of high and low objective risk were defined as follows^10^: *High objective risk* if the women had a higher risk than the mean risk of women at the same age. *Low objective risk* if the women had an identical or lower risk than the mean risk of women at the same age.

#### Perceived comparative risk

In face-to-face interviews, women were asked about their risk compared with other women of the same age. Perceived comparative risk was assessed with the question “How do you classify your risk of breast cancer compared with other women of your own age?” Perceived comparative risk was measured on a seven-point Likert scale that ranged from “much lower” to “much higher,” respectively. Perceived comparative risk was defined as follows: *High perceived comparative risk* if women responded “a little higher,” “higher,” and “very much higher,” respectively. *Low perceived comparative risk* if women responded “very much lower,” “lower,” “a little lower,” and “the same,” respectively.

#### Estimated comparative risk

To define the *estimated comparative risk*, the categories of objective risk and perceived comparative risk were related to each other. *Estimated comparative risk* was categorized according to Banegas et al. as follows^11^: *Accurate*, if women with high objective risk had a high perceived comparative risk and if women with low objective risk had a low perceived comparative risk. *Underestimate*, if women with high objective risk had a low perceived comparative risk. *Overestimate*, if women with low objective risk had a high perceived comparative risk.

### Statistical analysis

Pearson's chi-square (*χ*^2^) test was applied to compare categorized variables. *t*-test, Mann–Whitney test (*U*-test) and analysis of variance (ANOVA) were applied to compare continuous variables. Results of multinomial logistic regression were presented as adjusted odds ratios (ORs), 95% confidence interval (95% CI), and *p*-value. *p*-Values of regression analyses were calculated using likelihood ratio tests for each variable. The four outcomes of regression analysis were defined by the performance of MS: never, sometimes, each year, and each second year. No performance of MS served as reference group. Significant variables of univariate regression analysis were used for regression modeling. Variables with significance level <0.2 in the univariate analysis were entered into the model. Then, variables with significance level <0.05 were kept in the model. Backward selection was used when significant variables were selected. The final model was tested for fitness using the likelihood ratio test. Statistical analysis was performed using SPSS STATISTICS™ software (SPPS; IBM company; version 24).

## Results

Altogether, data were obtained from 396 women (Table 1). Mean age ranged from 48.94 (SD = 12.10) years for women who never underwent MS, to 60.95 (SD = 9.89) years for women who underwent MS each second year (*p* = 0.000; Table 1). Of all 108 women who never underwent MS, 75 (69.44%) were between 40 and 49 years old (*p* = 0.000; Table 1). Seventy-three of 108 (67.59%) who never underwent MS and 70 of 99 (70.71%) who sometimes underwent MS had a low educational level (*p* = 0.005). Of 108 and 168 women who never underwent MS and underwent it every year, 13 (12.04%) and 41 (24.40%), respectively, had a family history (*p* = 0.008; Table 1). Women with low perceived comparative risk tended more often to perform MS never and sometimes (Table 1). Women with high perceived comparative risk in contrast performed MS more often during each year (Table 1). Of all 340 and 56 women with low and high perceived comparative risk, 138 (40.59%) and 34 (60.71%), respectively, performed MS during each year (*p* = 0.040; Table 1). Of all women aged between 40 and 49 years, 46 (32.39%) out of 142 and 15 (60.00%) out of 25 with low and high perceived comparative risk, respectively, performed MS during each year (*p* = 0.026; Table 1).

**Table 1.** Socioeconomic Variables and Perceived Comparative Risk Distributed According to Women's (*n* = 396) Participation in Mammography Screening

	Never (n = 108)	Sometimes (n = 99)	Each year (n = 168)	Each second year (n = 17)	p

	48.94 (SD = 12.10)	57.74 (SD = 11.90)	55.58 (SD = 10.74)	60.95 (SD = 9.89)	0.000
	*n* (%)	*n* (%)	*n* (%)	*n* (%)	
Age					
Mean					
40–49 years	75 (44.91)	31 (18.56)	61 (36.53)	—	0.000
50–59 years	13 (12.04)	32 (32.32)	51 (30.36)	6 (28.57)	
60–69 years	9 (8.33)	17 (17.17)	38 (22.62)	5 (23.81)	
≥70 years	11 (10.19)	19 (19.20)	22 (13.09)	6 (28.57)	
Education					
Low	73 (67.59)	70 (70.71)	98 (58.34)	9 (42.86)	0.005
Middle	29 (26.85)	25 (25.25)	54 (32.14)	6 (28.57)	
High	6 (5.56)	4 (4.04)	16 (9.52)	6 (28.57)	
Marital status					
No union	40 (62.96)	37 (37.37)	67 (39.88)	8 (38.10)	0.963
Stable union	68 (37.04)	62 (62.63)	101 (60.12)	13 (61.90)	
Ethnic origin					
Caucasian	72 (66.67)	65 (65.66)	106 (63.10)	13 (61.90)	0.923
Not Caucasian	36 (33.33)	34 (34.34)	62 (36.90)	8 (38.10)	
Income					
Low	75 (69.44)	62 (62.63)	103 (61.31)	9 (42.86)	0.373
Middle	30 (27.78)	33 (33.33)	57 (33.93)	10 (47.62)	
High	3 (2.78)	4 (4.04)	8 (4.76)	2 (9.52)	
Occupation status					
Occupied	31 (28.70)	35 (52.70)	36 (21.43)	8 (38.10)	0.061
Not occupied	77 (71.30)	34 (47.30)	132 (78.57)	13 (61.90)	
Family history of breast cancer					
No	95 (87.96)	89 (89.90)	127 (75.60)	17 (80.95)	0.008
Yes	13 (12.04)	10 (10.10)	41 (24.40)	4 (19.05)	
Perceived comparative risk					
All women (*n* = 396)					
Low	96 (28.24)	90 (26.47)	138 (40.59)	16 (4.70)	0.040
High	12 (21.43)	9 (16.07)	34 (60.71)	1 (1.79)	
Women aged 40–49 years (*n* = 168)					
Low	67 (47.18)	29 (20.42)	46 (32.39)	—	0.026
High	8 (32.00)	2 (8.00)	15 (60.00)	—	
Women aged ≥50 years (*n* = 229)					
Low	29 (14.65)	61 (30.81)	92 (46.46)	16 (8.08)	0.434
High	4 (12.9)	7 (22.58)	19 (61.29)	1 (3.23)	

SD, standard deviation.

During the interviews, women were also asked about limitations and benefits of MS. When women were asked if MS can prevent breast cancer, 357 (90.15%) responded correctly that it does not prevent the disease. Asked about the potential of MS to lower risk of death because of breast cancer, 215 (54.29%) responded that it does not lower risk of death. Of these 215 women who believed that MS cannot lower risk of death, 75 (34.88%) never performed MS and 140 (65.12%) performed it (*p* = 0.000).

Univariate analysis indicated that participation in MS varied among age groups (*p* = 0.000; Table 2). Women aged 40–49 years participated 2.6 times less often every year (OR = 0.380; 95% CI: 0.17–0.85), compared with women aged ≥70 years (Table 2). Women with low and middle educational level participated 8.1 times less often (OR = 0.123; 95% CI: 0.33–0.47) and 4.8 times less often (OR = 0.207; 95% CI: 0.05–0.87), respectively, in biannual MS, compared with women with high educational level (Table 2). Furthermore, women with no family history of breast cancer had a 2.4 (OR = 0.424; 95% CI: 0.22–0.84) times reduced chance of participation in annual MS compared with women with family history (Table 2). Occupation status had borderline significance (*p* = 0.061), whereas heterogeneous data distribution of marital status, income, and ethnic origin was insignificant (Table 2).

**Table 2.** Unadjusted Odds Ratio and Confidence Intervals of Women Who Performed Mammography Screening (MS) (*n* = 288) Are Presented for Single Socioeconomic Variables in Univariate Analysis. Women Who Did Not Participate in MS (*n* = 108) Served as Reference Group

	n (%)	Sometimes (n = 99)	Each year (n = 168)	Each second year (n = 21)	p
		OR (95% CI)	OR (95% CI)	OR (95% CI)	
Age					
40–49 years	167 (42.17)	0.239^*^ (0.10–0.56)	0.407^*^ (0.18–0.90)	**—**	0.000
50–59 years	102 (25.76)	1.43 (0.53–3.81)	1.962 (0.76–5.05)	0.846 (0.21–3.39)	
60–69 years	69 (17.42)	1.094 (0.37–3.28)	2.111 (0.76–5.89)	1.019 (0.23–4.47)	
≥70 years	58 (14.65)	Ref.			
Education					
Low	250 (63.13)	1.438 (0.39–5.32)	0.503 (0.19–1.35)	0.123^*^ (0.33–0.47)	0.021
Middle	114 (28.79)	1.293 (0.33–5.11)	0.698 (0.25–1.98)	0.207^*^ (0.05–0.87)	
High	32 (8.08)	Ref.			
Marital status					
No union	152 (38.38)	1.015 (0.58–1.78)	1.128 (0.69–1.86)	1.046 (0.40–2.74)	0.963
Stable union	244 (61.62)	Ref.			
Ethnic origin					
Caucasian	140 (35.35)	1.046 (0.59–1.86)	1.170 (0.70–1.95)	1.231 (0.47–3.24)	0.923
Mixed ethnicity	256 (64.65)	Ref.			
Income					
Low	249 (62.88)	0.620 (0.13–2.88)	0.515 (0.13–2.01)	0.713 (0.18–2.89)	0.399
Middle	130 (32.83)	0.825 (0.17–3.99)	0.180 (0.03–1.23)	0.500 (0.07–3.44)	
High	17 (4.29)	Ref.			
Occupation status					
Occupied	110 (27.78)	0.736 (0.41–1.32)	1.476 (0.85–2.58)	0.654 (0.25–1.73)	0.062
Not occupied	286 (72.22)	Ref.			
Family history of breast cancer					
No	328 (82.83)	1.218 (0.51–2.92)	0.424^*^ (0.22–0.84)	0.582 (0.170–1.10)	0.008
Yes	68 (17.17)	Ref.			

^*^ *p* ≤ 0.05.

CI, confidence interval; OR, odds ratio.

Associations between risk estimates and MS attendance are summarized in Table 3. In univariate analysis, the women with low perceived comparative risk had a twofold (OR = 0.493; 95% CI: 0.24–1.00) decreased chance to participate in MS annually, compared with women with high perceived comparative risk (*p* = 0.020; Table 3). Women aged from 40 to 49 years with low perceived comparative risk had a 2.7 (OR = 0.366; 95% CI: 0.14–0.93) times decreased chance of annual MS attendance (*p* = 0.027; Table 3).

**Table 3.** Association of Perceived and Estimated Comparative Risk with Adherence to the Mammography Screening Program

Age group	n (%)	OR_CRUDE_ (95% CI)			p	OR_ADJUSTED_ (95% CI)			p
		Sometimes	Each year	Each second year		Sometimes	Each year	Each second year	
Low perceived comparative risk^a^									
All women	340 (85.86)	1.250 (0.50–3.11)	0.493^*^ (0.24–1.00)	2.500 (0.31–20.34)	0.020	1.044 (0.39–2.79)	0.578 (0.26–1.26)	3.146 (0.33–30.41)	0.143
40–49 years	142 (84.52)	1.731 (0.35–8.66)	0.366^*^ (0.14–0.93)	—	0.027	1.076 (0.20–5.84)	0.386 (0.13–1.16)	—	0.184
≥50 years	198 (86.46)	1.202 (0.33–4.44)	0.668 (0.21–2.12)	2.207 (0.23–21.46)	0.407	1.060 (0.26–4.31)	0.721 (0.21–2.51)	2.139 (0.17–26.67)	0.690
Overestimation of comparative risk^b^									
All women	39 (9.85)	0.444 (0.10–2.06)	0.406 (0.11–1.52)	0.089 (0.080–1.029)	0.017	0.632 (0.13–3.13)	0.577 (0.15–2.23)	0.158 (0.01–2.10)	0.419
40–49 years	16 (9.58)	0.333 (0.03–4.19)	0.381 (0.06–2.53)	—	0.186	0.237 (0.02–3.27)	0.548 (0.07–4.11)	—	0.601
≥50 years	23 (10.04)	0.667 (0.08–5.54)	0.510 (0.07–3.51)	0.167 (0.01–2.82)	0.453	0.703 (0.8–6.13)	0.513 (0.07–3.70)	0.275 (0.01–5.52)	0.749
Accurate estimation of comparative risk^b^									
All women	317 (80.05)	0.437 (0.13–1.50)	0.227^*^ (0.08–0.68)	0.126^*^ (0.03–0.52)	0.017	0.592 (0.16–2.15)	0.382 (0.12–1.19)	0.268 (0.06–1.22)	0.419
40–49 years	140 (83.83)	0.403 (0.05–3.01)	0.196^*^ (0.04–0.99)	—	0.186	0.309 (0.04–2.53)	0.350 (0.06–1.92)	—	0.601
≥50 years	177 (77.29)	0.667 (0.13–3.52)	0.340 (0.07–1.57)	0.214 (0.03–1.33)	0.453	0.623 (0.12–3.35)	0.353 (0.08–1.67)	0.285 (0.04–1.96)	0.749

Data of women who underwent mammography (*n* = 288) are presented as ORs and CIs in univariate analysis; (OR_CRUDE_) and adjusted (OR_ADJSUTED_) for age, education level, occupation status, and family history of breast cancer in multivariate analysis. Women who never underwent mammography (*n* = 108) served as reference group.

^a^ High perceived comparative risk served as reference group.

^b^ Underestimation of comparative risk served as reference group.

^*^ *p* ≤ 0.05.

Of all women aged between 40 and 49 years, respectively, ≥50 years, 16 (9.58%) and 23 (10.04%) overestimated the comparative risk (*p* = 0.117; Table 3). Altogether, women who overestimated comparative risk had an 11.2 (OR = 0.089; 95% CI: 0.080–1.029) times decreased chance to perform MS each second year (*p* = 0.017; Table 3). Those women who estimated comparative risk accurately had a 4.4 (OR = 0.227; 95% CI: 0.08–0.68) and 7.9 (OR = 0.126; 95% CI: 0.03–0.52) times decreased chance to perform MS each year and each second year, respectively, compared with women who underestimated the risk (*p* = 0.017; Table 3).

Perceived comparative risk was dependent on family history and this effect was stronger in women aged ≥50 years compared with those aged between 40 and 49 years (Table 4). Women without family history of breast cancer, aged between 40 and 49 years, and ≥50 years, had a 5.8-fold (OR = 0.173; 95% CI: 0.07–0.44) and 9.3-fold (OR = 0.108; 95% CI: 0.05–0.25) decreased chance to have a high perceived comparative risk, compared with women with family history (*p* = 0.000; *p* = 0.000; Table 4).

**Table 4.** The Chance to Have a High Perceived Comparative Risk (*n* = 56) Is Shown for Women Who Have No Family History of Breast Cancer in Univariate Analysis

High perceived comparative risk				
		n (%)	OR_CRUDE_ (95% CI)	p
Family history of breast cancer				
All women^a^	No	328 (82.83)	0.132^a^ (0.07–0.25)	0.000
40–49 years	No	139 (83.20)	0.173^a^ (0.07–0.44)	0.000
≥50 years	No	189 (82.53)	0.108^a^ (0.05–0.25)	0.000
	Yes		Ref.	

ORs and 95% CIs are shown for all women (*n* = 328) without family history of breast cancer, and women aged between 40 and 49 years (*n* = 142), respectively, ≥50 years (*n* = 189), without it. Low perceived comparative risk served as reference group.

^a^ Adjusted for age.

Sociodemographic variables and risk estimates of univariate analysis both were used for models of regression analysis (Table 5). Risk estimates did not contribute to models of independent variables. In the final model, women with low educational level had an 11.4-fold (OR = 0.088; 95% CI: 0.02–0.37) decreased chance to perform MS each second year (*p* = 0.040; Table 5). Women who were not occupied had a 2.3-fold (OR = 0.427; 95% CI: 0.22–0.82) and 3.2-fold (OR = 0.314; 95% CI: 0.10–1.01) decreased chance to perform MS sometimes, respectively, each second year (*p* = 0.010; Table 5). Finally, women without family history had a 2.2-fold (OR = 0.454; 95% CI: 0.23–2.50) decreased chance to perform MS each year (*p* = 0.022; Table 5).

**Table 5.** An Age-Adjusted Model of Nominal Logistic Regression Representing All Women (*n* = 396)

	n (%)	MS			p
		Sometimes (n = 99)	Each year (n = 168)	Each second year (n = 21)	
		OR (95% CI)			
Education					
Low	250 (63.13)	1.252 (0.32–4.83)	0.417 (0.15–1.19)	0.088^*^ (0.02–0.37)	0.040
Middle	114 (28.79)	1.616 (0.39–6.62)	0.839 (0.28–2.50)	0.278 (0.06–1.26)	
High	32 (8.08)	Ref.			
Occupation status					
Not occupied	286 (72.22)	0.427^*^ (0.22–0.82)	1.132 (0.62–2.06)	0.314^**^ (0.10–1.01)	0.010
Occupied	110 (27.78)	Ref.			
Family history					
No	328 (82.83)	1.213 (0.49–2.98)	0.454^*^ (0.23–2.50)	0.614 (0.17–2.23)	0.022
Yes	68 (17.17)	Ref.			

ORs and CIs are presented for women who underwent MS (*n* = 288) in multivariate analysis. Women who did not perform MS (*n* = 108) served as reference group.

^*^ *p* < 0.050; ^**^p = 0.053.

MS, mammography screening.

## Discussion

Brazilian women aged 40–49 years are exposed to conflicting public recommendations regarding the benefit of MS. As the Ministry of Health recommends an age threshold of 50 years, it was not surprising that women aged 40–49 generally had lower participation rates of MS, compared with older women. Notably, more than 90% of interviewed women knew that MS cannot prevent breast cancer, however, more than half of all 396 women denied that it can lower the risk of dying from breast cancer. This indicated that most women also did not consider MS to be beneficial. Of the 215 women who believed that MS does not lower risk of death, most performed it nevertheless. One factor could be that many women rely on recommendations of their physician independent of their own opinion. Another factor could be that women, even those who believe that MS does not lower chance of death, perform it to know if they are healthy.

To the best of our knowledge, this is the first Brazilian study to analyze women's perception of estimation of comparative risk and its impact on MS behavior. Low perceived comparative risk was associated with a decreased chance of MS participation during each year. Similarly, a recent study associated perceived low risk with decreased participation in MS.^24^ However, when the perceived risk was associated with the objective risk of women, then the resulting estimated comparative risk tended to be inversely associated with MS participation. In univariate analysis, overestimation and accurate estimation of comparative risk were negatively associated with MS participation. This was surprising, as most previous studies did not reveal any positive or negative association of comparative risk estimation with participation in MS.^10,12,16,20^ Labrie et al.^24^ suggested that fear of breast cancer increased the perception of personal risk among women aged 30–49 years. A recent study performed in Malta suggested that nonadherence to organized MS programs was associated with fear.^25^ Similarly, women in the present study who feared the disease might have tended to overestimate risk had an accurate risk estimation. In addition, regular participation in MS could also lead to underestimation of risk.

Present data suggest that the perception of comparative risk was dependent on family history of breast cancer. In women aged ≥50 years, this effect was more prominent than for women aged 40–49 years. In a Turkish study, perception of risk did not depend on family history.^14^ By contrast, similar to our results, other studies performed in Turkey, Canada, and the United States revealed a positive association between family history of breast cancer and comparative risk perception.^12,18,26^

After adjustment of data, risk estimates did not contribute to heterogeneous distribution of MS categories in models of regression analysis. Education level, occupation status, and family history instead were identified as independent variables. Women without family history had a reduced chance of participation in regular MS. This result is in agreement with previous Brazilian studies that reported decreased MS attendance among women without family history of cancer.^9,27^ Similarly, studies from Iran and the United States reported a positive association between family history of breast cancer and adherence to MS programs.^18,20^

Data suggest that women with lower educational levels tended to undergo MS less often. In recent studies from various countries, high educational level was one of the most important predictors of adherence to MS.^28–31^ In agreement with our results, several Brazilian studies reported a greater chance of adherence to MS among women with higher educational levels.^5,8,27,32,33^ In contrast, a recent study also performed in the Northeast region of Brazil, among women who had predominantly a low educational level, high income instead of education was associated with MS participation.^9^ The nationwide adherence study by Narayan et al. in the United States identified both high income and educational level as important predictors of adherence to MS.^29^

Data of the present study also indicated that occupied women performed MS more often compared with those who were not occupied. A positive association between performance of MS and occupation was also found in previous studies.^34,35^ A recent Australian study, in contrast, revealed that among women with home duties, the chance to perform ever a mammogram was higher, compared with women with professional or technical occupation.^36^ The relationship between occupation and MS performance may be highly dependent on the social context.

Our study had several limitations: Participants were randomly selected. However, a selection bias that favored a certain socioeconomic background and a defined risk estimation of breast cancer cannot be excluded. Data sampling in a health service center may have caused a selection bias toward women with health seeking behavior. As only women with breast and ovarian cancer were excluded from the study, sampling may have caused a bias toward individuals with chronic diseases. Estimated risk was determined as a categorical rather than as a continuous variable. This may have obscured the power of the study to detect more detailed associations with MS behavior. Results of our study cannot necessarily be extrapolated to other regions of the country, particularly as income was an important variable in several previous Brazilian studies. Low resolution of data may have been linked to small sample size. Finally, the study did not elucidate why women who overestimated comparative risk, respectively, estimated it accurately and participated less often in MS, compared with women who underestimated it.

## Conclusions

Neither perception nor estimation of comparative risk was independent variables that explained women's MS behavior. Educational level, occupation status, and family history instead were the most important variables explaining heterogeneity of women's MS behavior. Perception of high comparative risk was associated with family history. Overestimation and accurate estimation of breast cancer risk did not lead to an increase of MS performance. Fear could be an important psychological variable that determines both overestimation and avoidance behavior. It might be desirable for health authorities to provide more detail regarding the benefits and limits of MS. More than half the women did not understand that MS is capable of reducing the risk of death from breast cancer. Conflicting opinions regarding its benefit are probably confusing women aged 40–49 years. Prospective Brazilian studies regarding the benefit of MS are lacking, and it is to date unclear whether MS can decrease the mortality rate of breast cancer among Brazilian women.

## Abbreviations Used

ANOVA: analysis of variance
CI: confidence interval
INCA: National Cancer Institute
MS: mammography screening
NIH: National Institute of Health
OR: odds ratio
SD: standard deviation
